# Will I die of coronavirus? Google Trends data reveal that politics determine virus fears

**DOI:** 10.1371/journal.pone.0258189

**Published:** 2021-10-06

**Authors:** Joan C. Timoneda, Sebastián Vallejo Vera

**Affiliations:** 1 Department of Political Science, Purdue University, West Lafayette, Indiana, United States of America; 2 Department of Political Science, Tecnologico de Monterrey, Mexico City, Mexico; Politechnika Wroclawska, POLAND

## Abstract

Is Google Trends (GT) useful to survey populations? Extant work has shown that certain search queries reflect the attitudes of hard-to-survey populations, but we do not know if this extends to the general population. In this article, we leverage abundant data from the Covid-19 pandemic to assess whether people’s worries about the pandemic match epidemiological trends as well as political preferences. We use the string ‘will I die from coronavirus’ on GT as the measure for people’s level of distress regarding Covid-19. We also test whether concern for coronavirus is a partisan issue by contrasting GT data and 2016 election results. We find strong evidence that (1) GT search volume close matches epidemiological data and (2) significant differences exist between states that supported Clinton or Trump in 2016.

## Introduction

There are three promising avenues of research for political scientists using Google Trends (GT) –a service by Google that aggregates search data by input term, geographical location, and time. First is surveying populations. Chykina and Crabtree [1] aptly show how specific search terms reflect the preoccupations of certain groups of people. Their example is the search term “Will I be deported?”, which only people susceptible to deportation use. A second role for GT in research is generating alternative proxies for useful variables. Chadwick and Şengül [2] show evidence that searches for ‘unemployment’ in Turkey closely mirror the unemployment rate (see also [3, 4] for similar applications). The third application for GT is forecasting, which has been subject of long and inconclusive academic research [5–10]. Timoneda and Wibbels [11] argue that incorporating variance in GT search interest can help us forecast protests.

This paper focuses on the first avenue of research, namely, the potential for GT to serve as an alternative to survey populations. Since the publication of Chykina and Crabtree’s [1] piece, few works have expanded on their findings or probed whether they apply to different populations or issues. We do just that. Taking advantage of abundant data around the COVID-19 pandemic, we analyze searches on Google in different American cities and states and ask the following questions: can GT tell us the extent to which people are worried about the coronavirus? And, more importantly, are these worries created by high levels of cases and deaths in these locations or are they politically motivated?

We expect people to become worried about the virus on two grounds: epidemiology and politics. First, if people see increases of cases and deaths in their state, they will become more concerned about the virus. Second, if they believe the virus to be a general health threat, they will be more concerned. We find evidence that GT searches for ‘will I die from coronavirus’ are highly correlated with both political preferences as well as coronavirus cases and deaths. This is a strong indication that GT data can be used to survey the general population regarding their level of concern for the virus. Interestingly, we find that there are strong differences along party lines in how people become concerned about the virus, confirming that the pandemic has indeed become a partisan issue. States where Clinton’s vote share was high in 2016 tend to show greater concern about the coronavirus, while states where she lost are significantly less concerned. Conversely, the correlation between people’s worries about the virus and the actual number of cases and deaths is weaker than the relationship between worries about the virus and partisanship across US states. With these findings, this article provides two takeaways for current and future research. First, confirming Chykina and Crabtree’s [1] main intuition, GT can be used effectively to survey populations, provided the search terms used are representative. Second, and more substantively, political cleavages are more likely to determine people’s attitudes toward certain social phenomena than factual evidence. This finding is important given today’s polarized political climate and matches well with other results in the literature on polarization.

## Using Google Trends to survey populations

Chykina and Crabtree [1] use a search string that only people who may be affected in the present or in the future are likely to use. In their article, the search string of choice is ‘will I get deported’, as only people who are at risk of being deported are likely to use this phrasing on Google’s search engine. They show that searches for ‘will I get deported’ coincide with key immigration moments such as Trump’s travel ban in early 2017 or Arizona’s passing of a restrictive ‘Safe Neighborhoods’ bill in 2010. Spikes in search interest occurred mostly in areas with large immigrant populations such as New York, California and Texas. More broadly, other works has shown a strong correlation between search volume for a given term and changes in real world trends in epidemiology, health, finance, and political referenda [12–18].

We opted for ‘will I die from coronavirus’. Drawing from Chykina and Crabtree’s [1] strategy, we consider that the future tense helps identify worry, while the singular form of the first person indicates that the Google user is primarily concerned about their own well-being. While Chykina and Crabtree [1] focused on hard-to-survey immigrant populations, our population of interest is everyone who is at risk of contracting coronavirus. We thus attempt to extend Google’s power to survey beyond specific groups and into the general population. Also, our choice of a strong word such as ‘die’ over a more generic one, say ‘get’, is rooted in the need to select strings that can capture as precisely as possible the attitudes of the people being surveyed. A search term like ‘will I get coronavirus’ may capture worry, but it may also capture people who simply want to estimate the likelihood of being sick, but are not overly worried about the implications for their health –as with ‘will I get the flu’ searches every year. Our string captures people’s worries about the virus and its long-term health effects well.

The preceding discussion points to a key aspect of using GT for surveying populations: search strings must be carefully considered, include a tense in the singular form of the first person, and use terms that precisely isolate the attitude or sentiment on which we seek to survey people. GT will always generate a certain amount of error –we can never know precisely why people searched for what they did–, but we can (1) minimize the amount of noise and (2) and ensure that the error left is mostly white noise by carefully selecting our search strings.

## Data, approach and descriptive results

The most abundant and geographically precise GT data are in the United States. They are available at the country, state, metro area and city level, while in most other countries these data are only systematically available and accessible at the second administrative level (states or their equivalent). This is the first reason to focus this research on the US experience with the coronavirus. Two others follow. First, the US has been hard-hit by the pandemic and has both the highest levels of cases and deaths in the world as of this writing. Second, the country is highly polarized politically, and the COVID pandemic has also been subject of heated partisan debate. The United States’ erratic response to the crisis, in terms of lack of federal mandates and guidelines as well as wide state-to-state variation, is largely due to this fact [19, 20].

Our sample consists of all 50 US states. For each, we collect GT data for the time frame between February 18 and May 30 of 2020, which captures the initial peak of the pandemic of around late March, the period before the pandemic hit, and the weeks in April when the virus curve started to decline. The data are for two simultaneous search strings: ‘will I die from coronavirus’ and ‘will I die’. For every time unit (days) within the period, GT produces two index scores between 0 and 100, one for each search string. In the entire period only one score of 100 will exist and will be given to the day/search term that registered the highest search volume. The rest of the scores will be indexed proportionally to the day with the highest score (see S1 Appendix for further explanation of how GT’s algorithm works. Please see [11] for additional information on how GT produces the data researchers can use). Given the way GT’s algorithm works, including a parallel generic search term such as ‘will I die’ in the data collection process helps us benchmark results for ‘will I die from coronavirus’ across different states. Without the parallel term, each city’s data would operate within an independent index range between 0 and 100, making cross-city comparisons difficult. We consider (and the data bear this out) that ‘will I die’ is steady over time and there are few reasons to expect different states to have large disparities in search volume. Sample code is included in S1 Appendix.

**Fig 1:**
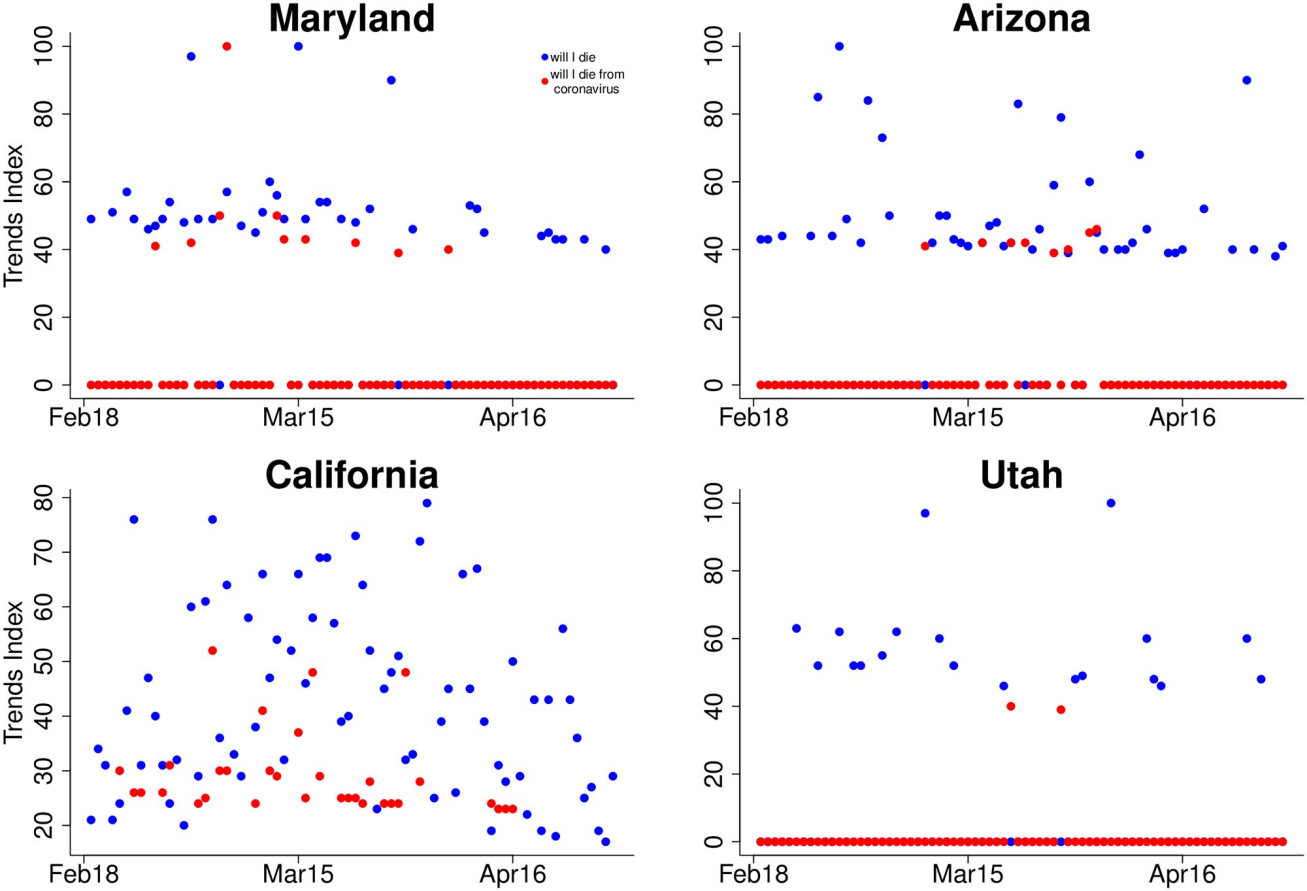
‘will I die’ (blue) vs. ‘will I die from coronavirus’ (red). Same GT collection.

The code returns daily data for this three-month period for each of the geographical units introduced earlier. Fig 1 shows the results for four selected states. Two of these states skew liberal (Maryland and California) while two of them lean conservative (Arizona and Utah). Relative to ‘will I die’ searches, ‘will I die from coronavirus’ searches are much more frequent in Maryland and California than they are in Arizona and Utah, where interest peaks during the second half of March and then becomes marginal by the time the virus peaked in early April. We thus begin to see some clear differences in how worried people are about the coronavirus across different states. But it could be that these differences are created by the level of incidence of the virus in each state, that is, where there are more cases and deaths, people tend to be more worried. This is consistent in the case of California, one of the the hardest-hit city in the early days of the pandemic and where people searched for ‘will I die from coronavirus’ more consistently.

To determine whether incidence of coronavirus in a state plays a role, or the extent of its role, on people’s searches for ‘will I die from coronavirus’, we collected data on COVID-19 infections and deaths for each of the 50 US states. The data are from Johns Hopkins University and are widely available at the county, state, and national level from different sources (the data can be accessed at https://github.com/CSSEGISandData/COVID-19 or https://doi.org/10.1016/S1473-3099(20)30120-1.) We use a count for total new cases and deaths per day by state. In our analysis, we first provide descriptive evidence for the association between partisanship and search volume for ‘will I die from coronavirus’ on Google. Then we model the probability that ‘will I die from coronavirus’ generates high volume conditional on whether the state voted for Trump or Clinton in 2016.

**Fig 2:**
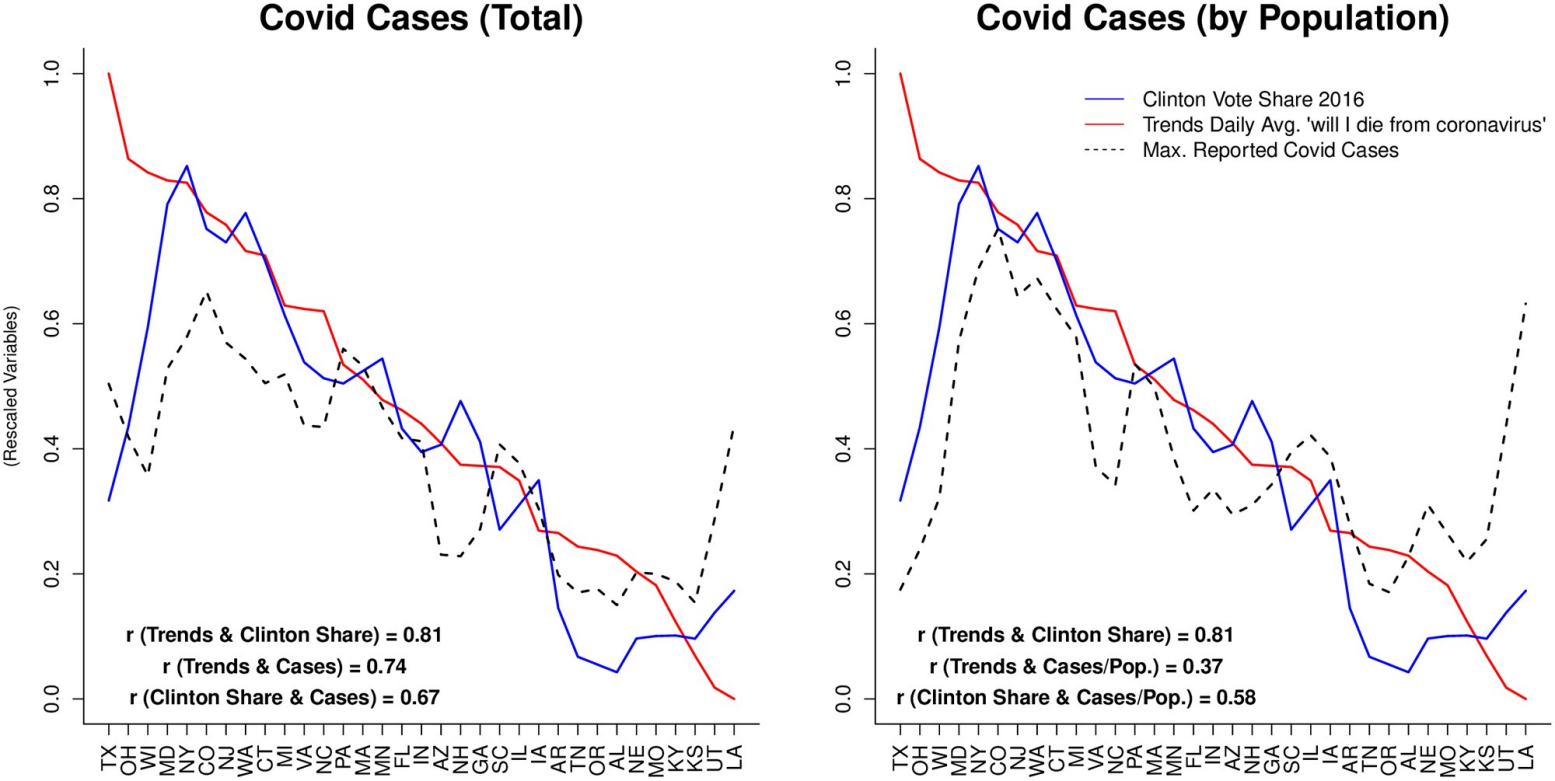
Is GT useful as a surveying tool? (I).

Fig 2 (and Fig 2A in S1 Appendix) provides strong evidence that GT is an effective tool for surveying populations. The red line represents the aggregate sum of search interest for ‘will I die from coronavirus’ for each state during the two and half month period under study (GT will only provide data for searches that exceed a certain threshold –see S1 Appendix for a discussion on this issue). All states have been sorted on the GT variable for the plot. Note that we limit our sample to states where the search volume for our term exceed the minimum threshold at least once, i.e. have one daily non-zero score over this period. Small states whose search volume is low tend to have a Trends index of 0 because searches never reach the minimum threshold set by Google (see S1 Appendix for further explanation). These states are: Alaska, Delaware, Hawaii, Idaho, Maine, Mississippi, Montana, Nevada, New Mexico, North Dakota, Oklahoma, Rhode Island, South Dakota, Vermont, West Virginia and Wyoming. These states are split more or less equally in terms of support for Trump or Clinton in 2016. Note also that California is missing from the figure as it is an outlier in GT searches. A figure with California is included in S1 Appendix. The blue line represents the state-wide vote share for Clinton in the 2016 general election. The dashed black line represents the total number of reported cases in Fig 2 (and the total number of deaths in Fig 2A in S1 Appendix). We provide the results for cases and deaths normalized by population (right plots) and without normalization (left plots). We provide the normalized results of Covid data by population because GT data is also normalized by population automatically by Google. There are two main takeaways from the Fig 2. One is that coronavirus hit democratic states much harder than republican ones, as widely reported at the beginning of the pandemic and likely due to faster spread in urban areas (See https://www.economist.com/graphic-detail/2020/05/22/covid-19-is-hitting-democratic-states-harder-than-republican-ones). Clinton vote share and total coronavirus cases are correlated at 0.67 for the period under study. The second takeaway is that, despite this initial disparity in the spread of coronavirus, the correlation between search interest in ‘will I die of coronavirus’ and Clinton 2016 vote share is much stronger (0.81) than the other correlations. The *r* score for searches on GT and total number of total cases stands at 0.72, and is much lower for GT searches and cases when normalized over population (0.37). It is also much stronger than the aforementioned 0.67 correlation between Clinton vote share and cases. Results for deaths are similar, with an *r* score of 0.74 for total cases and 0.36 for normalized cases (see S1 Appendix). Fears of the coronavirus on GT are thus highly correlated with political preferences (Clinton’s 2016 vote share) and Covid-19 cases and deaths. This is strong evidence that GT reflects sentiments toward the coronavirus.

## Modeling the probability of high state-wide searches for ‘Will I die of coronavirus’

The results hint at the possibility that politics, not epidemiology, better explain fears of the coronavirus. This is in line with other novel research [21–27] and the fact that the US government’s response to the pandemic became highly politicized in 2020. To further explore this hypothesis, we model the probability that ‘will I die from coronavirus’ registers activity on GT conditional on whether Trump or Clinton won the state’s electoral votes in the 2016 general election. We code the dependent variable as 1 if a state registered a search volume greater than 0 for ‘will I die from coronavirus’ in a given day and 0 otherwise. The data from GT, therefore, are again aggregated at the level of the state and are available daily (the unit of observation is the state-day). The reason we dichotomize the variable and opt for a logistic model is that GT data are not normally distributed, with zero-inflation and a relatively uniform distribution of positive values between 1 and 100. The results are unaffected is we use cut-points other than 0. For instance, 32 is the mean trends index score if zeros are removed. If we select 32 as our cut-point, the model remains significant. It remains significant up until the 75th percentile of the non-zero trends index distribution, a score of 41 (note that the results are unchanged using an OLS model). Hence, the outcome is whether a state registered positive activity for ‘Will I die of coronavirus’ on a given day. We control for three important potential confounders. One is state-level population density, as the virus spreads faster in urban areas which in turn are more likely to support Democrats. Second, we control for the state-level unemployment rate in April of 2020. Third, we account for state-level demographics by adding the percent of urban versus rural population as a control. Note that while we use daily level data, temporal dependence is not a substantial concern in the analysis. Covid cases are positively and highly correlated with time, as expected, but GT scores are not. Lastly, to account for regional differences in the spread of the virus and GT scores in the US, we add region fixed effects (The regions are the Northeast, Midwest, South and West.) We also conduct a series of sensitivity analyses to ensure that the model is robust to unobserved confounders and model specification (see S1 Appendix). The model is as follows:

$$\begin{array}{cc} P{\left(TS\right)}_{0,1} & =\alpha +\beta_{1}*log\left(covid\_cases\right)+\beta_{2}*clinton\_won+\beta_{3}*log\left(covid\_cases\right)*clinton\_won+\\ & \;\beta_{4}*log{\left(covid\_cases\right)}^{2}*clinton\_won+pop\_density+unemployment+\\ & \;pct\_urban+region_{i}+\epsilon \end{array}$$ (1)

**Fig 3:**
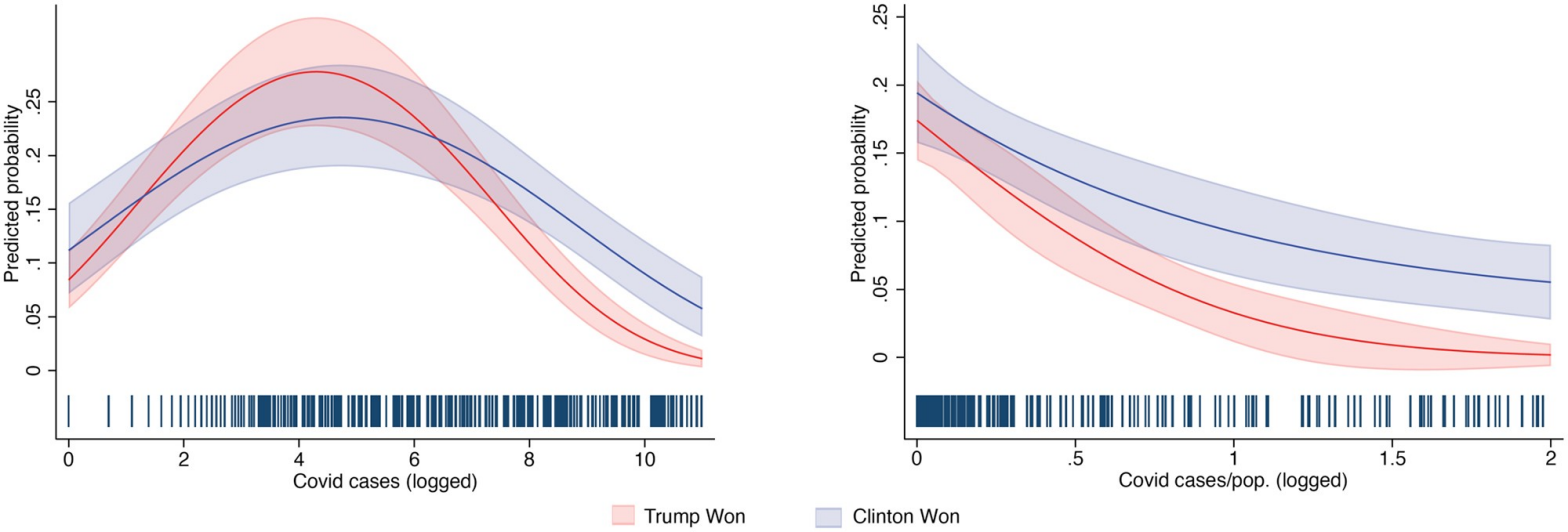
Whence the fear, politics or epidemiology? (V). *Note*: N = 3,650; Log Lik. = -1107.12.

The variables for Covid cases and deaths have been logged, and we use a quadratic term to capture non-linearity in the relationship (The results obtain if we use one single parameter and up to four polynomials.) The model interacts these Covid-related variables with a dichotomous variable for whether Clinton carried a given state in 2016. We use the same model for Covid deaths and provide the results also for cases and deaths normalized by population in Fig 3 and Fig 3A in S1 Appendix. The main results are in Fig 3 (see Table A1 in the S1 Appendix for the full results). The y-axis represents the predicted probability of observing substantial search volume on ‘will I get coronavirus’ as Covid-19 cases and deaths increase in states that Trump and Clinton won in 2016. At low levels of cases and deaths, differences among the two groups are not statistically significant. In fact, for both groups, predictions at low levels of *x* tend to be at their highest points, which can be explained by the fact that people’s worries initially are less partisan, as people begin to inform themselves about the novel coronavirus and become worried.

The situation reverses as the numbers of confirmed infections and deaths increase. While search volume for ‘will I die from coronavirus’ decreases slightly in Democratic-leaning states, the decline is sharp in those that supported Trump in 2016. The difference becomes statistically significant once the number of diagnosed cases surpasses 1096 (unlogged). The difference is starkest where deaths are concerned: while people in states that supported Clinton remain equally worried about dying from coronavirus as deaths increase in their state, people in states that supported Trump become significantly less likely to be concerned after 99 deaths have been registered in their state (We discuss the number for total deaths and cases without normalization. We present the normalized results for reference.)

There are three further considerations we need to address for our results to be sustained. One is related to how information spreads and why, how, and when people decide to Google ‘will I die of coronavirus’. Overall, the data show that people are more likely to use this search string (1) early on in the pandemic when information is scarce and (2) when deaths around them are high, conditional on their politics not impeding their assessment of risk. Our results stand on solid ground here, as people continue to search for this string as the pandemic evolves. Yet we should further research the ‘lifespan’ of certain terms as it relates to their ability to survey populations, considering that their use falls as people become better informed. Second, our choice to use data at the state level may raise questions regarding whether national or city-level data matter, too. They do. People inform themselves in the national and local news and use that information to evaluate risks. The state offers the best compromise between proximity to the user of Google’s search engine and data availability on GT. Our aim is to extend the present study to the level of the metro-area, with the expectation that increased proximity to new cases and deaths will exacerbate people’s concerns about the virus.

Lastly, people could search ‘will I die from coronavirus’ because they have poor health insurance. Thus, they worry about lack of access to healthcare should they catch it, not about the virus itself. While plausible, this explanation cannot be driving our results. People in states with larger urban areas and better-paying service jobs, which on average have the best insurance plans, should be *less* concerned about dying from the virus, not more. Since Clinton carried a large majority of the urban vote in 2016, health care would be biasing our results downward, not upward.

The results show strong support for the two main objectives of this research note. One is that GT can be used to survey populations, and we certainly obtain relevant information regarding people’s concerns at the outset of the coronavirus crisis. Google search volume matches up nicely with data on partisanship and data on the Covid-19’s spread. Second, we provide evidence that people’s fears of Covid-19 are strongly influenced by their political beliefs.

## Supporting information

S1 Appendix This appendix includes further information on the Google Trends algorithm, further multiple robustness checks, and a suite of sensitivity analyses. (PDF)
