# Supplementary material for: Will I die of coronavirus? Google Trends data reveal that politics determine virus fears
Source: PLoS One. 2021 Oct 6;16(10):e0258189. doi: 10.1371/journal.pone.0258189 (PMC8494313; doi:10.1371/journal.pone.0258189)
Supplement: S1 Appendix — (PDF) [file pone.0258189.s001.pdf]

Appendix A1

1. How Google Trends Works

Google Trends (GT) is a service provided by Google that gathers data on search volume for given terms, or text strings, within a specific location and time. Researchers can select whichever string they want to find information on, without limitations. We selected 'will i die from coronavirus' in this article, and Chykina and Crabtree (2018) used 'will I get deported'. Terms can have one or more words, depending on the question we want to answer, but it is recommended in general to minimize the amount of words to maximize search volume. In our case, because we want to survey a population, it is necessary to add 'will i' to the beginning of our string, but other researchers have used only one word –for instance, Timoneda and Wibbels (2020) forecast protests using the word 'protest'.

As for time, we can obtain data from 2004 onward for whichever period we want. In our case, we opted for a two and a half month window between February 18 and April 30. Note that if the search window is under a week, the data is aggregated by Google at the hourly level; between 1 week and 9 months, daily; between 9 months and 5 years, weekly; and over 5 years, monthly. In our case, because our time frame is 2.5 months, we obtain daily trends data.

Researchers can also set the location for their collection. The obvious starting point is to do a worldwide search, which returns global popularity for a text string at a given time. Then, the level of geographic specificity of the data varies by country. In some places, data are only available at the country level. This is true for some countries in Africa where there is too little volume to generate sufficient regional scores. Most countries have at least some data at the first administrative level – states, regions or the equivalent. This is the level we use in this paper because it represents a good compromise between proximity to the Google user and large amounts of search volume that maximize variation in the data. Lastly, in countries such as the United States, there is a large amount of GT data available at metro area level.

How is the GT index score produced? Google does not release the raw percentage that a given search string represented within a period of time. This is proprietary and only health researchers can obtain access to these data. While some applications in political science may be linked to health outcomes, this is rather rare. For this reason, it is important that we learn to use Google's primary index score in productive ways. Google creates this 'index score', which ranges from 0 to 100, based on the percentage of overall search volume that a given string represented within a period (which only Google knows). First, their algorithm finds out when a given string was most popular within the requested time frame.

In more technical terms, it assigns a value of 100 to the data point (hours, days, weeks, or months, depending on the length of the collection) that represented the *highest* share of overall traffic on their search engine. That's when the string was 'most popular' within a given period. Once that score of 100 is assigned, the rest of the scores are assigned proportionally to the highest score. If the term was half as popular at another time point, that data point is assigned a score of 50. By 'half as popular' we mean that it represented half of the overall search traffic as compared to the peak data point. This strategy by Google will guarantee that a score of 100 exists (assuming the overall search is popular enough to register in the first place). An example: say, for instance, that the term 'coronavirus' accounted for 5% of all Google traffic on March 15, and that we are collecting between March 1 and March 30 (hence the data will be daily). Assume also that 'coronavirus' did not represent more than 5% of overall traffic in other day in March. March 15 is, therefore, the most popular day, and thus it receives a score of 100. As researchers, the only score we would know is 100 for March 15; we would never know that it represented 5% of overall traffic. Say, then, that on March 26, 'coronavirus' represented 2.5% of all traffic on Google. Since 2.5 is half of 5, we would observe a GT index score of 50 for March 26. If volume decreased to 1% on March 30, we would observe a score of 20.

Now to another difficult wrinkle to comprehend: If a term does not register sufficient volume on a given day but there is sufficient volume to produce a trend for the entire period, that one day where volume was too low will receive a score of 0. Say that everyone forgot about coronavirus on March 24 (they did not) –in that case, GT would assign a score of 0 to that day, not because volume represented over 100 times less than the 5% from March 15, but because there isn't sufficient volume to even register. What this means in practice is that any day that has 100 times less than the volume of the peak day is confounded with any day that does not register even a single hit (in the extreme). In practice, it is hard to determine whether this affects results, generally speaking. We suspect that it does not in our case, but further research is needed to establish this for certain. What is certain is that Google also does not release this 'minimum threshold' for a search term to be considered viable for GT purposes. If we refer to 'low volume' searches at any point in the paper, we are referring to this issue.

Finally, it is important to mention that the results we obtain from GT for each one of our queries are based on a *sample* drawn by Google. The sample is from overall search traffic for the period and location specified and, according to Google, is unbiased. This means that doing multiple search of the same term over time will produce slightly different samples.[1] While the results remain using different samples of the same search term, no two samples are identical. Future work must explore how this

[1]See `https://support.google.com/trends/answer/4365533?hl=en` for more information regarding the sampling procedure.

variation affects the research using on GT data.

Below is an example of one of the collections for the San Francisco metro area (using Python's `pytrends` package):

```
data = pred.build_payload([‘will I die’, ‘will I die from coronavirus’],
                                timeframe='2020-02-18 2020-05-15',
                                geo='US-CA-807')
```

2. Terms and States

As discussed in the main text, we follow Chykina and Crabtree (2018) and use the search term 'will i die from coronavirus'[2] as a proxy for issue salience. One limitation to GT data is 'low volume' searches. Our data is at the state level, so we aim to use a search term that 1) captures the question of interest (e.g. how worried are people about the coronavirus) and 2) yields the least number of 'low volume' searches. In addition to 'will i die from coronavirus', we gathered data for the following terms: 'will i die coronavirus','will i die from the coronavirus', and 'will i die from corona'. The U.S.-wide trend for each search term is presented in Figure 1A. For all four coronavirus-related searches, the pattern is alike. There is a peak on searches around March 12 that slowly declines all through April. This suggests that all of these terms are capturing a similar sentiment about the coronavirus at a similar time.

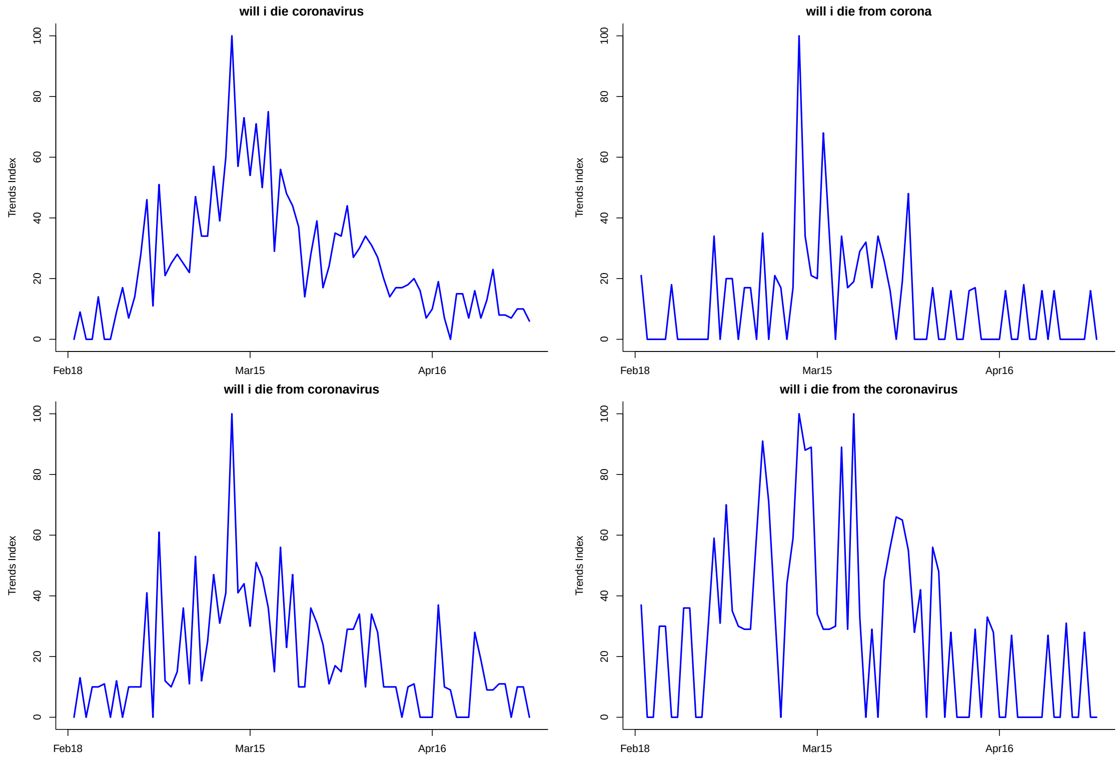

Figure 1A: Google Trends index for different search terms in the United States between February 15 and April 30 2020.

[2]Searches in GT are case insensitive and exclude punctuation.

However, of these four terms, ‘will i die from coronavirus’ has the lowest number of states with ‘low volume’ searches for the entirety of the period analyzed (i.e. do not have at least one daily non-zero score over this period). While the other three search terms have, at least, 26 states with ‘low volume’ searches, our ‘will i die from coronavirus’ search term produce only 14 states with ‘low volume’ searches. These states are: Alaska, Delaware, Hawaii, Idaho, Maine, Mississippi, Montana, North Dakota, Oklahoma, Rhode Island, South Dakota, Vermont, West Virginia and Wyoming. Including these states, which went for Trump and Clinton in more or less equal measure, could lead to misleading results. States with smaller populations are less likely to generate the enough search volume to pass the threshold used by Google to provide results. This does not necessarily mean that there is no interest in the topics explored, or that the interest is constantly low. It just means that the raw volume of searches, a condition linked to the size of the population rather than to the interests of their users, is not high enough to make it into the data set. Artificially setting their search rate to zero would bias our results introducing values that are not reflective of the actual variation of interest across time.

Researchers must be observant of these limitations when choosing search terms, finding the right balance between lexical accuracy and the volume of data recovered. Future work should explore what are the consequences of using different terms and the how these relate to the actual interest of people.

3. Full table

The table below reports the full results for our model:

	(1) Trends
Cases	0.642***
	(0.093)
Cases × Cases	−0.075***
	(0.010)
Clinton	0.412
	(0.281)
Clinton × Cases	−0.282*
	(0.131)
Clinton × Cases × Cases	0.037**
	(0.014)
Pop. Density	0.001**
	(0.000)
Unemployment	0.073***
	(0.019)
Constant	−3.654***
	(0.327)
Observations	2482
Pseudo R^2	0.057
Log-Lik.	−976.613

	(2) Trends
Deaths	0.320*
	(0.125)
Deaths × Deaths	−0.083***
	(0.023)
Clinton	0.377*
	(0.190)
Clinton × Deaths	−0.277
	(0.160)
Clinton × Deaths × Deaths	0.062*
	(0.027)
Pop. Density	0.001*
	(0.000)
Unemployment	0.072***
	(0.018)
Constant	−2.985***
	(0.288)
Observations	2482
Pseudo R^2	0.037
Log-Lik.	−976.613

Table A1: Full results for main model (Figure 1).

4. Additional Tests

We present additional tests that further illustrate the relation in interest on the coronavirus and coronavirus deaths and cases, and the differences across states where Clinton and Trump won in the 2016 elections. In Figure 2A we divided our sample between states where Clinton won in the general election in 2016, and states where Trump won. For each group, we correlate the number of coronavirus-related deaths (left panel) and cases (right panel) with the GT index. The solid lines represent the GT trend and the dashed line represents coronavirus deaths/cases. The results show that the correlation between GT and death/cases is higher for states where Clinton won (0.68 and 0.66 respectively) than for states where Trump won (0.41 and 0.38 respectively). We further explore the details of this relation in the main paper.

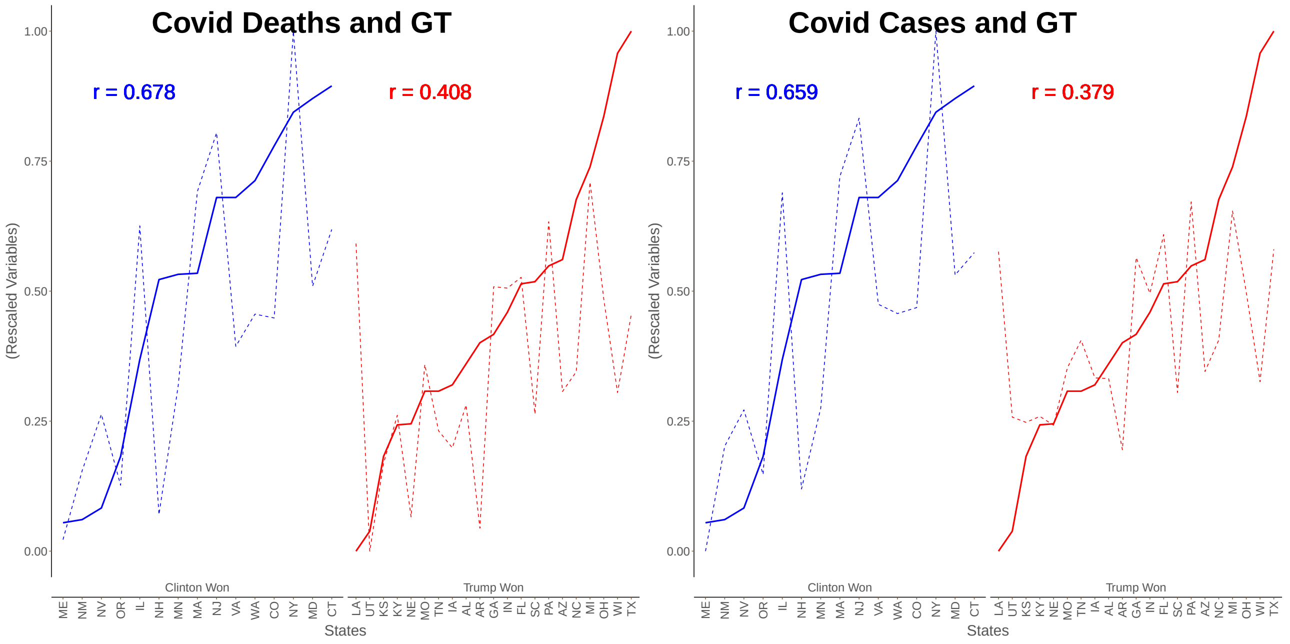

Figure 2A: Correlation between GT and number of deaths (left panel) and cases (right panel). States divided into two groups: those where Clinton won in the 2016 elections, and those where Trump won.

5. Figures 3A and 4A (Covid Deaths)

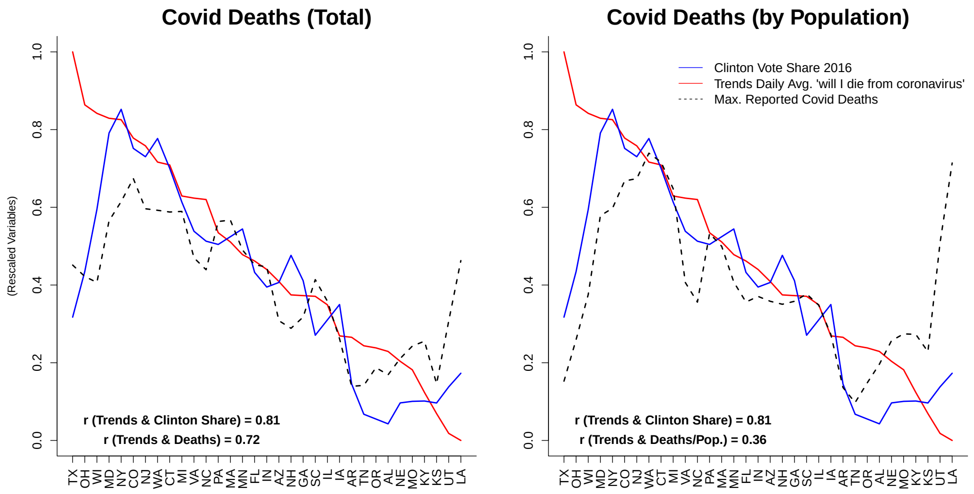

Figure 3A: Is GT useful as a surveying tool? (II)

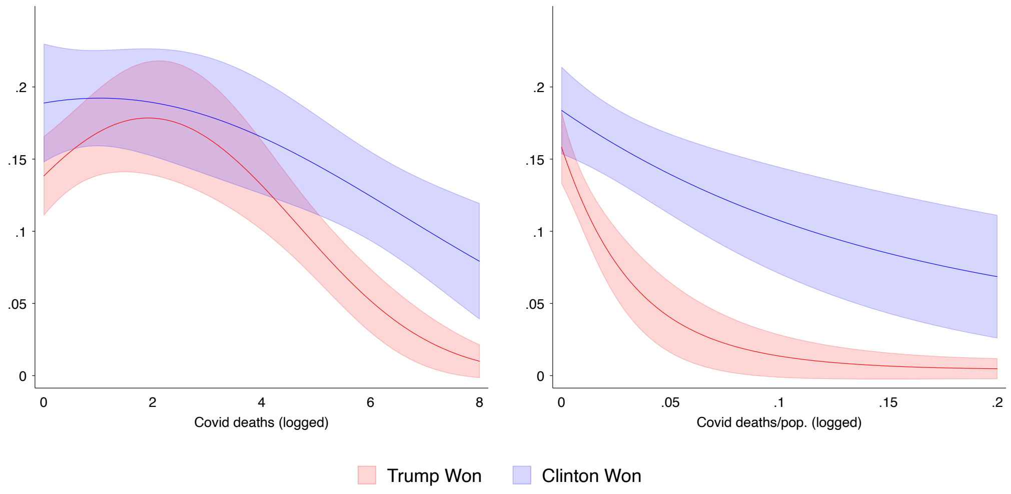

Figure 4A: Whence the fear, politics or epidemiology? (V)
Note: N = 3,650; Log Lik. = -1107.12.

6. Sensitivity Analysis

In order to check for bias-inducing unobserved confounders, we implement a set of sensitivity checks, as developed by Cinelli and Hazlett (2020). Since Cinelli and Hazlett's (2020) approach is designed for linear models, we estimate a linear probability model that replicates our main results (see Table A1). Both the results and the conclusions hold for this new specification (see Table A3 and Figure 5A). From this model (and from our main model), we are interested in the robustness of our interaction term $ClintonVotexCases^2$. We want to know how strong an unobserved confounder has to be in order to affect the significance and direction of our variable of interest (and, thus, our main conclusions). We bound the plausible strength of the unobserved confounders to our variable *density*, a strong determinant for the use of the search term "will i die from coronavirus." The robustness value for bringing the point estimate of $ClintonVotexCases^2$ exactly to zero ($RV_{q=1}$) is 4.5%. This means that unobserved confounders that explain 4.5% of the residual variance of *both* the dependent variable and $ClintonVotexCases(log)^2$ are required to explain away all the observed effect.

DV: *Trends*

IV:	Est.	S.E.	t-value	$R^2_{Y\sim D\|\mathbf{X}}$	$RV_{q=1}$	$RV_{q=1,\alpha=0.05}$
$ClintonVotexCases^2$	0.003	0.001	2.299	0.2%	4.5%	0.7%
df = 2470		*Bound (1x density)*: $R^2_{Y\sim Z\|\mathbf{X},D} = 0.3\%$, $R^2_{D\sim Z\|\mathbf{X}} = 1.1\%$				

Table A3: Sensitivity statistics for $ClintonVotexCases^2$.

To better understand the relation, we present sensitivity contour plots (see Figure 5A) that show what is the necessary strength of a confounder variable with a hypothetical strength of the variable *density*. The plot (left panel of Figure 5A) reveals that the effect of our variable is robust to a confounding variable up to seven times as strong as the variable *density*. For the effect of our $ClintonVotexCases^2$ variable to stop being statistically significant at the 0.95 level, the confounding variable would need to have 1.2 times the effect of the *density* variable at explaining the dependent variable *and* our $ClintonVotexCases^2$ (see right panel of Figure 5A). It needs to be 2.5 times greater to lose significance at the 0.9 level.

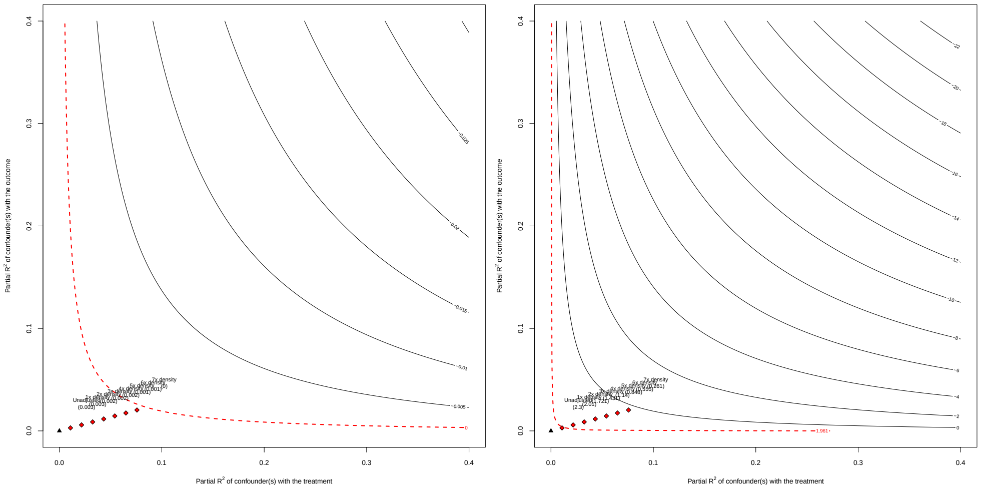

Figure 5A: Sensitivity contour plots for variable $ClintonVotexCases^2$. In the left panel, the horizontal axis shows the hypothetical residual share of variation of the variable of interest $ClintonVotexCases^2$ that unobserved confounders explain. The vertical axis shows the hypothetical partial R2 of unobserved confounders with the dependent variable $Trends$. The contours show what would be the estimate for $ClintonVotexCases^2$ that one would have obtained in the full regression model including unobserved confounders with such hypothetical strengths. The right panel shows the sensitivity of the $t-value$.

7. Extreme Bounds Analysis

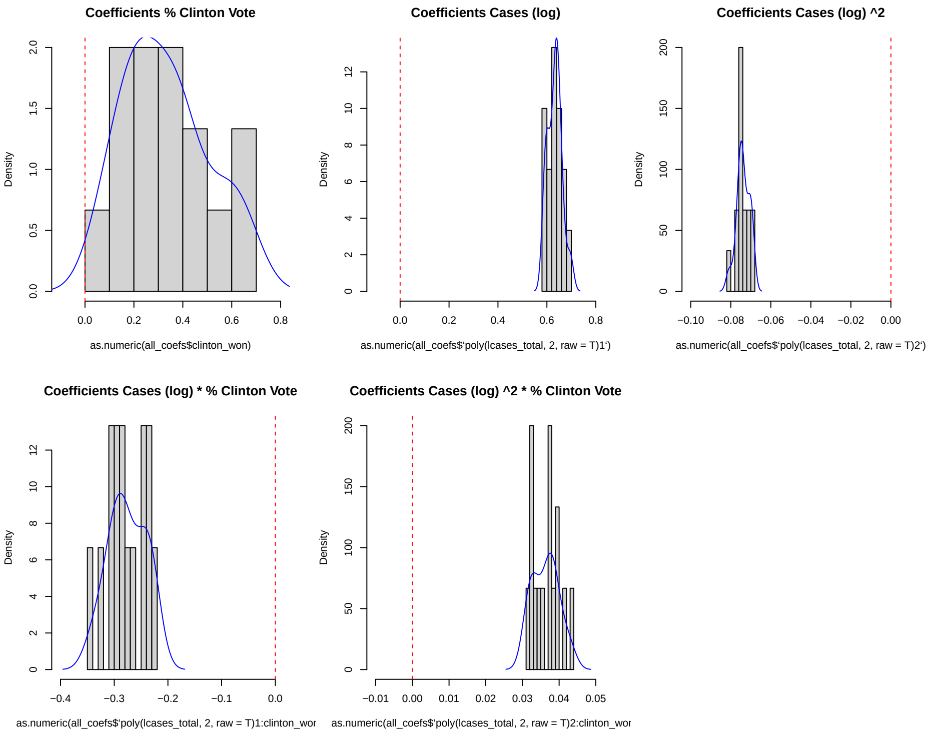

Figure 6A: Distribution of coefficients from EBA on variables of interest

In order to partially assess the sensitivity of our results and address possible specification problems, we perform an extreme bounds analysis (EBA) on our main model (see Table A1). EBA examines how robustly our dependent variable, *Trends*, is associated with our determinants of interest (*Cases* and *Clinton*). To achieve this, we run a total of 15 regressions with all the possible combination of control variables in our model. Thus, we keep in all our regressions the variables in our main interaction, *Cases* and *Clinton*, and vary the controls. We present the results of the distribution of the coefficients of our variables of interest in Figure 6A.

Following Sala-i-Martin (1997), we calculate the CDF(0)–i.e.,the cumulative density function evaluated at zero–for our variables of interest. The coefficients of the variables *Cases*, and *Cases*-squared x *Clinton*, have 95% of the CDF(>0); the coefficients of the variables *Cases*-squared, *Cases* x *Clinton* have 95% of the CDF(<0); and only the coefficients of the variable *Clinton* has 85% of the CDF(>0) which is to be expected from the main model. We show the EBA results in Table A4.

	Trends														
	**Model 1**	**Model 2**	**Model 3**	**Model 4**	**Model 5**	**Model 6**	**Model 7**	**Model 8**	**Model 9**	**Model 10**	**Model 11**	**Model 12**	**Model 13**	**Model 14**	**Model 15**
Clinton	0.341	0.602**	0.419	0.326	0.657**	0.195	0.247	0.544*	0.082	0.412	0.102	0.251	0.380	0.207	0.186
	(0.302)	(0.268)	(0.284)	(0.286)	(0.272)	(0.281)	(0.295)	(0.290)	(0.294)	(0.289)	(0.293)	(0.286)	(0.301)	(0.296)	(0.296)
Clinton x Cases	−0.302**	−0.293**	−0.270**	−0.233*	−0.341***	−0.266**	−0.235*	−0.320**	−0.223*	−0.282**	−0.243*	−0.309**	−0.285**	−0.246*	−0.292**
	(0.130)	(0.127)	(0.127)	(0.129)	(0.128)	(0.129)	(0.129)	(0.128)	(0.129)	(0.130)	(0.130)	(0.130)	(0.130)	(0.129)	(0.131)
Clinton x Cases x Cases	0.039***	0.038***	0.036***	0.032**	0.043***	0.035***	0.032**	0.041***	0.031**	0.037***	0.032**	0.039***	0.037***	0.033***	0.037***
	(0.013)	(0.013)	(0.013)	(0.013)	(0.013)	(0.013)	(0.013)	(0.013)	(0.013)	(0.013)	(0.013)	(0.013)	(0.013)	(0.013)	(0.013)
Cases	0.695***	0.590***	0.595***	0.600***	0.637***	0.618***	0.600***	0.638***	0.637***	0.642***	0.621***	0.662***	0.641***	0.646***	0.662***
	(0.093)	(0.092)	(0.092)	(0.092)	(0.092)	(0.093)	(0.092)	(0.092)	(0.092)	(0.092)	(0.093)	(0.093)	(0.092)	(0.093)	(0.093)
Cases x Cases	−0.081***	−0.069***	−0.070***	−0.070***	−0.074***	−0.072***	−0.070***	−0.075***	−0.074***	−0.075***	−0.073***	−0.077***	−0.075***	−0.075***	−0.077***
	(0.010)	(0.010)	(0.010)	(0.010)	(0.010)	(0.010)	(0.010)	(0.010)	(0.010)	(0.010)	(0.010)	(0.010)	(0.010)	(0.010)	(0.010)
Pop. Density	−0.001**			0.001***			0.001***			0.001***	0.0004		0.001**	−0.001**	0.0003
	(0.001)			(0.0003)			(0.0004)			(0.0003)	(0.0003)		(0.0004)	(0.001)	(0.0003)
Unemployment	0.085***				0.078***			0.077***		0.073***		0.075***	0.075***		0.073***
	(0.020)				(0.019)			(0.020)		(0.019)		(0.019)	(0.020)		(0.019)
Urban	0.062***					0.039***			0.047***		0.037***	0.039***		0.060***	0.037***
	(0.009)					(0.006)			(0.007)		(0.007)	(0.007)		(0.009)	(0.007)
Region: Northeast	0.534**		0.464**				0.120	0.343*	0.215				0.043	0.569**	
	(0.247)		(0.200)				(0.245)	(0.203)	(0.206)				(0.240)	(0.253)	
Region: South	0.409**		0.121				0.045	0.180	0.186				0.120	0.292*	
	(0.172)		(0.159)				(0.161)	(0.161)	(0.160)				(0.163)	(0.168)	
Region: West	−0.626***		0.152				0.237	0.104	−0.364*				0.195	−0.604***	
	(0.228)		(0.191)				(0.194)	(0.194)	(0.204)				(0.198)	(0.228)	
Constant	−8.316***	−2.481***	−2.580***	−2.607***	−3.616***	−5.433***	−2.666***	−3.702***	−6.060***	−3.654***	−5.276***	−6.447***	−3.769***	−6.939***	−6.311***
	(0.774)	(0.175)	(0.199)	(0.180)	(0.333)	(0.527)	(0.201)	(0.363)	(0.580)	(0.333)	(0.541)	(0.595)	(0.365)	(0.697)	(0.613)
N	2482	2482	2482	2482	2482	2482	2482	2482	2482	2482	2482	2482	2482	2482	2482

***p < .01; **p < .05; *p < .1

Table A4: Full results for extreme bounds analysis.

8. Figure 2 with outliers (California in Google Trends variable)

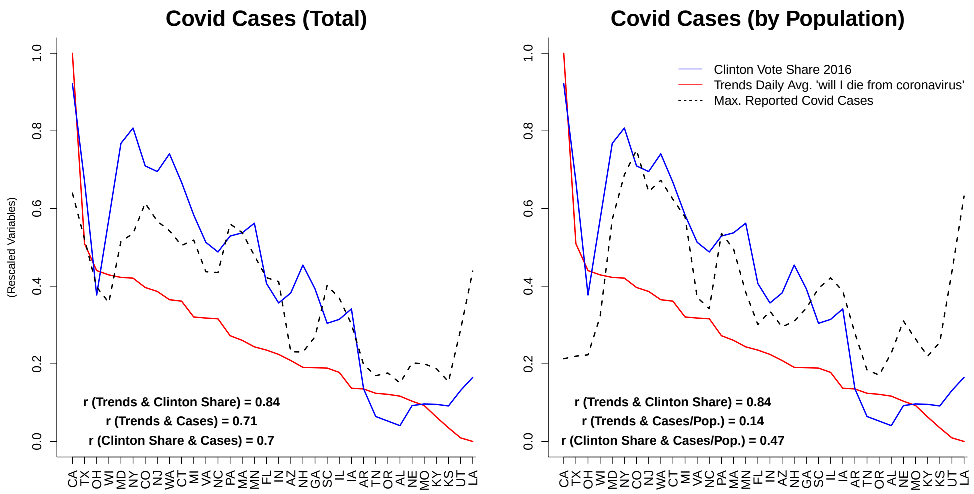

Figure 7A: Figure 2 (main paper) with California
